# Pediatric nurses’ stress and their knowledge, attitudes, and practices towards first-aid for pediatric trauma: a latent profile analysis

**DOI:** 10.1080/07853890.2026.2696066

**Published:** 2026-07-04

**Authors:** Yuyan Sun, Huimin Zhang, Huanhuan Wang, Huifang Jiao, Dandan Li, Caixiao Shi

**Affiliations:** a Surgical Intensive Care Unit, Henan Children’s Hospital [Zhengzhou Children’s Hospital], Zhengzhou, China; b School of Nursing, Ningxia Medical University, Ningxia, China; c School of Nursing, Henan Medical University, Xinxiang, China; d Nursing Department, Henan Children’s Hospital [Zhengzhou Children’s Hospital], Zhengzhou, China

**Keywords:** Pediatric nurses, perceived stress, first aid, pediatric trauma, knowledge, attitudes, practices, latent Profile analysis, stress management

## Abstract

**Background:**

To explore the perceived distress and knowledge, attitudes, and practices (KAP) regarding first-aid for pediatric trauma among pediatric nurses.

**Material and Methods:**

A cross-sectional study was conducted between May and August 2025 at four tertiary hospitals in Henan Province and included pediatric nurses. The Perceived Stress Scale (PSS-14) was used to assess perceived distress and perceived coping. A structured questionnaire was used to evaluate KAP regarding pediatric trauma first-aid. Latent profile analysis (LPA) was applied to identify distinct stress profiles. KAP scores >70% were considered good.

**Results:**

The study included 472 pediatric nurses. The knowledge, attitude, and practice scores were 44 [40,46] (80% of maximum), 35.5 [32,38] (88.8% of maximum), and 29.04 ± 5.81 (72.6% of maximum), respectively, suggesting good KAP. The LPA identified four profiles: Profile 1 (high perceived distress + high perceived coping, 26.48%), Profile 2 (moderate perceived distress + moderate perceived coping, 47.88%), Profile 3 (moderate perceived distress + low perceived coping, 15.25%), and Profile 4 (low perceived distress + moderate perceived coping, 10.38%). The Profile 1 reported the highest perceived distress [24 (23, 25)] and the best perceived coping abilities [15 (13, 17)]. Consequently, this profile consistently scored highest in knowledge [44 (42, 49)] and attitude [37 (33, 39)], suggesting that effective coping mechanisms may buffer the negative effects of high stress.

**Conclusions:**

This study highlights the diverse stress profiles among pediatric nurses and their associations with pediatric trauma first-aid. The results emphasizing the importance of targeted interventions to enhance coping mechanisms. Future training programs should consider stress management strategies alongside skill development.

## Introduction

According to World Health Organization data, pediatric trauma accounts for over 950,000 deaths annually among children under 18 years old, with 95% occurring in low- and middle-income countries. In developing regions, trauma-related mortality rates are 3.4 times higher than in developed countries [1]. Children are especially vulnerable to unintended accidents and injuries due to their developing bodies and limited awareness of environmental hazards [2,3]. Timely and appropriate first aid intervention within the “golden hour” following pediatric trauma can reduce mortality by up to 30%. As primary first responders, nurses play a crucial role in delivering this time-sensitive care, with their actions directly impacting patient outcomes. However, the unique anatomical and physiological characteristics of children, such as smaller airways, higher body surface-to-mass ratio, and limited compensatory mechanisms, require specialized knowledge and skills for effective emergency response [4,5].

The Knowledge, Attitude, and Practices (KAP) framework provides essential insights into healthcare providers’ preparedness for emergency care. Evidence shows that knowledge gaps in pediatric trauma care can lead to delayed recognition of critical conditions, inappropriate interventions, and increased complications [6–8].

Studies indicate that inadequate knowledge of pediatric trauma assessment tools can result in missed injuries in up to 25% of cases, while proper training can improve accurate injury identification by 40%. Additionally, nurses’ attitudes toward pediatric trauma care significantly impact their willingness to engage in continuing education and protocol adherence [9,10]. Pediatric nurses experience unique stressors in their work beyond typical emergency care demands, including managing distressed parents, performing painful procedures on children, and dealing with potential long-term developmental impacts of trauma. These work-related stressors contribute to overall perceived stress levels that may impact KAP scores. Research shows that high stress levels can impair clinical decision-making by up to 30% and increase medication error risks by 20%. These challenges are particularly pronounced in pediatric trauma care, where precise dosing and quick reactions are crucial [11,12]. Emerging evidence suggests that psychological attributes such as emotional intelligence may enhance clinical decision-making in high-acuity pediatric settings, with nurses demonstrating higher emotional intelligence showing superior clinical judgment in critical situations [13]. It suggests that the relationship between stress and KAP scores may be mediated by nurses’ psychological resources and coping capacities.

Previous research has employed the KAP framework to assess emergency care practices, revealing concerning gaps in first aid knowledge despite positive learning attitudes [14]. Among pediatric nurses, studies have identified particular gaps in trauma assessment tools and age-specific interventions. For example, research on NICU nurses in neighboring regions has documented significant knowledge deficits regarding evidence-based developmental care practices [15]. Yet the relationship between practice and distress remains unexplored. Studies from various regions have primarily focused on teachers and parents [16–18], while the relationship between perceived distress and KAP among pediatric nurses remains largely unexplored, particularly in the Chinese healthcare context.

Therefore, this study aimed to (1) identify distinct perceived distress profiles among pediatric nurses using latent profile analysis based on the PSS-14, (2) compare knowledge, attitudes, and practices regarding pediatric trauma first aid across the identified perceived distress profiles to examine their associations with practice, and (3) explore demographic and professional characteristics associated with profile membership.

## Methods

### Study design and participants

This cross-sectional study was conducted between May 10, 2025, and August 31, 2025, at four tertiary hospitals in Henan Province, and included pediatric nurses. The inclusion criteria were 1) pediatric nurses with a professional qualification certificate, 2) at least 1 year of work experience in a pediatric department, and 3) provided informed consent to participate in this study. The exclusion criteria were 1) nursing interns or 2) nurses on rotation assignments or on a level. This study was approved by the Ethics Committee of Henan Children’s Hospital (2025-IIT-007-002), and informed consent was obtained from all participants.

### Sample size

The sample size was determined using Cochran’s formula: *n*_0_ = *Z*^2^ × *p* × (1–*p*)/*e*^2^, where *n*_0_ represents the required sample size, *Z* is the critical value for a 95% confidence level (1.96), *p* is the estimated population proportion (set at 0.5 to maximize sample size), and *e* represents the margin of error (0.05) [19]. Using these parameters, the calculation yielded a minimum required sample size of 384 participants.

### Questionnaire

The KAP questionnaire was developed specifically for this study based on a comprehensive review of the literature and expert consensus regarding the early management of pediatric trauma emergencies. Following initial design, it was refined based on input from 3 senior experts in pediatric emergency nursing and subjected to a pilot test involving 44 participants, yielding a reliability coefficient (Cronbach’s α) of 0.8144 (0.8049 for knowledge, 0.8857 for attitude, and 0.7130 for practice). Specifically, the Cronbach’s α coefficient for the Perceived Stress Scale (PSS)-14 used in the pilot study was 0.7312, indicating acceptable internal consistency.

The finalized KAP questionnaire, administered in Chinese, comprises three sections: knowledge, attitudes, and practices. The knowledge section consists of eight questions encompassing 11 items (possible score range: 11–55 points), the attitude section includes eight questions (possible score range: 8–40 points), and the practice section contains five questions comprising eight items (possible score range: 8–40 points). Each item was rated on a five-point Likert scale. KAP scores >70% were considered good. The knowledge section employs a five-point Likert scale (e.g. “very knowledgeable” to “not knowledgeable at all”) to evaluate participants’ self-perceived knowledge rather than objective knowledge accuracy. This method captures respondents’ confidence and perceived familiarity with pediatric trauma concepts, rather than their actual factual understanding. Although this limits the ability to detect true knowledge gaps, it offers valuable insight into nurses’ perceived preparedness and potential training needs. Future studies should incorporate objective knowledge assessments with correct/incorrect scoring to validate these self-reported measures.

The PSS-14 was used to assess perceived distress levels, comprising 14 items rated on a five-point Likert scale (from 0 = never, 1 = almost never, 2 = sometimes, 3 = fairly often, to 4 = very often). The PSS-14 measures the degree to which situations in one’s life are appraised as unpredictable, uncontrollable, and overloaded. This instrument evaluates two distinct dimensions: perceived distress (measured through seven negatively worded items: 1, 2, 3, 8, 11, 12, 14) and perceived coping (assessed *via* seven positively worded items: 4, 5, 6, 7, 9, 10, 13). The PSS-14 scores are obtained by reverse scoring the seven positively stated items (items 4, 5, 6, 7, 9, 10, 13), where 0 = 4, 1 = 3, 2 = 2, 3 = 1, and 4 = 0, and then summing across all 14 items. The total PSS-14 score ranges from 0 to 56, with higher total scores indicating greater overall perceived distress. For the two subscales, negatively worded items (perceived distress subscale) are scored directly from 0 to 4, with higher scores indicating higher perceived distress (range: 0–28), while positively worded items (perceived coping subscale) require reverse scoring, with lower reversed scores indicating better perceived coping (range: 0–28). While the PSS-14 is not a diagnostic instrument and has no established clinical cut-off scores, general interpretive categories have been proposed: low perceived distress (0–18), moderate perceived distress (19–37), and high perceived distress (38–56) for the total score. However, in the present study, the two subscale scores were utilized separately in the latent profile analysis rather than the total score, as this approach allows for the identification of distinct patterns of distress and coping ability perceptions among participants. The Chinese version of PSS-14 has demonstrated strong psychometric properties in healthcare settings, with Cronbach’s alpha coefficients ranging from 0.78 to 0.83 and test-retest reliability of 0.85 over two weeks. Previous validation studies in Chinese nursing populations have confirmed its two-factor structure through confirmatory factor analysis (CFI = 0.92, RMSEA = 0.065), supporting the validity of analyzing the perceived distress and perceived coping ability dimensions separately [20].

The questionnaires were distributed by online method, and the pediatric nurses were invited to participate by a convenience sampling method. Convenience sampling was employed due to accessibility constraints and the need to achieve an adequate sample size within the study period, though this may limit generalizability, a point addressed in the limitations section.

### Statistical analysis

Statistical analyses were performed using SPSS 22.0 (IBM, Armonk, NY, USA) and Mplus 8 (Muthén & Muthén, Los Angeles, CA, USA). Continuous variables were tested for normality using the Shapiro-Wilk test. Variables with normal distribution were presented as mean ± standard deviation (Mean ± SD), while those with skewed distribution were reported as median with interquartile range, and categorical variables were expressed as frequencies and percentages (*n*, %). The normality of continuous variables was assessed using the Shapiro-Wilk test. For group comparisons, analysis of variance (ANOVA) was employed for normally distributed continuous variables, while the Kruskal-Wallis test was used for non-normally distributed data. Post hoc tests were conducted when significant differences were identified. Categorical data were compared using the chi-squared test or Fisher’s exact test, as appropriate. Spearman’s rank correlation analysis was performed to explore the relationships among knowledge, attitude, and practice scores.

Latent profile analysis (LPA) was conducted to identify subgroups of participants based on their responses to the PSS-14. The individual item scores of the PSS-14 served as manifest indicators in the LPA model. Models with one to four latent profiles were fitted, and the optimal number of profiles was determined using several model fit indices, including the Akaike Information Criterion (AIC), Bayesian Information Criterion (BIC), and sample-size adjusted BIC (aBIC). Entropy was calculated to assess classification accuracy, with higher values indicating better profile distinction. Statistical significance of model comparisons was further evaluated using the Lo-Mendell-Rubin likelihood ratio test and the bootstrap likelihood ratio test. Statistical significance was set at a two-tailed *P*-value of less than 0.05.

Following profile identification, between-profile comparisons were conducted to examine associations between profile membership and KAP outcomes. For continuous KAP variables, the Kruskal-Wallis test was used for non-normally distributed data (knowledge and attitude scores), while ANOVA was employed for normally distributed data (practice scores). Post hoc pairwise comparisons were performed when significant overall differences were detected to identify which specific profiles differed from one another. Chi-squared tests or Fisher’s exact test were used to compare categorical demographic and professional characteristics across profiles. These analyses allowed examination of how distinct perceived stress patterns (characterized by different combinations of perceived distress and perceived coping) were associated with KAP outcomes.

## Results

### Demographic characteristics and KAP scores

A total of 472 participants were included in this study, among them, 450 (95.34%) were female, with mean age of 34.84 ± 5.72 years, with mean 11.73 ± 6.13 years of work experience, 272 (57.63%) had intermediate professional title, 460 (97.46%) had fewer than 10 cases of prehospital first aid for pediatric trauma patients, 450 (95.34%) had fewer than 10 of emergency cases, 300 (63.56%) participated in training on pediatric trauma first aid, 427 (90.47%) were working in teaching hospital. The knowledge, attitude, and practice scores were 44 [40,46] (possible range: 11–55; 80.0% of maximum), 35.5 [32,38] (possible range: 8–40; 88.8% of maximum), and 29.04 ± 5.81 (possible range: 8–40; 72.6% of maximum), respectively, suggesting good KAP. Knowledge scores varied significantly by experience of prehospital first aid for pediatric trauma patients, experience of emergency treatment, training on pediatric trauma first aid (*p* < 0.001), organized trauma first aid, teaching hospital, and hospital with standardized first aid procedures. Attitude scores varied significantly by training on pediatric trauma first aid and teaching hospital. Practice scores varied significantly by experience of prehospital first aid for pediatric trauma patients, experience of emergency treatment, number of emergency cases, training on pediatric trauma first aid, organized trauma first aid, teaching hospital, and hospital with standardized first aid procedures (all of *p* < 0.05) (Table 1).

**Table 1.** Demographic characteristics and KAP scores.

*N* = 472	*n* (%)	Knowledge score		Attitude score		Practice score	
		Median (Q1, Q3)	P	Median (Q1, Q3)	P	Mean ± SD	P
Total score		44[40,46]		35.5[32,38]		29.04 ± 5.81	
Gender			0.406		0.062		0.497
Male	22(4.66)	43[39,45]		32.5[32,35]		29.86 ± 3.28	
Female	450(95.34)	44[40,46]		36[32,38]		29 ± 5.90	
Education			0.852		0.175		0.755
Associate degree or below	23(4.87)	44[38,51]		36[31,40]		29.78 ± 5.56	
Bachelor’s degree	431(91.31)	44[40,46]		35[32,38]		28.97 ± 5.84	
Master’s degree	18(3.81)	44[40,49]		37.5[36,39]		29.55 ± 5.58	
Doctorate	**/**						
Professional title			0.105		0.383		0.936
Junior level	171(36.23)	43[40,46]		35[32,38]		29.08 ± 5.82	
Intermediate level	272(57.63)	44[40,47]		36[32,39]		28.95 ± 5.96	
Senior level	13(2.75)	44[42,50]		35[33,37]		29.92 ± 5.20	
No professional title	16(3.39)	42[37,44]		34[31,38]		29.37 ± 3.24	
Monthly income, CNY			0.964		0.890		0.226
<5000	75(15.89)	44[38,46]		36[32,39]		29.68 ± 6.03	
5000–10000	324(68.64)	44[40,46]		36[32,38]		29.14 ± 5.59	
10000–20000	47(9.96)	44[42,46]		34[32,38]		27.51 ± 6.97	
Prefer not to disclose	26(5.51)	43.5[41,46]		35.5[32,37]		28.69 ± 5.34	
Experience of prehospital first aid for pediatric trauma			**<0.001**		0.120		**<0.001**
Yes	90(19.07)	44[44,50]		36[32,39]		31.8 ± 4.64	
No	382(80.93)	43[39,46]		35[32,38]		28.39 ± 5.87	
Experience with first aid			**<0.001**		0.386		**<0.001**
Yes	176(37.29)	44[42.5,48]		36[32,39]		30.50 ± 5.25	
No	296(62.71)	43[38,45]		35[32,38]		28.16 ± 5.96	
Number of first aid cases during the past year			0.143		0.416		**0.038**
Fewer than 10 cases	450(95.34)	44[40,46]		35.5[32,38]		28.89 ± 5.86	
10–50 cases	18(3.81)	44.5[42,49]		35[32,37]		31.55 ± 2.54	
50–100 cases	3(0.64)	44[39,45]		38[33,39]		32.66 ± 6.11	
100–200 cases	1(0.21)	53[53,53]		40[40,40]		40 ± 0	
More than 200 cases	/						
Experience of Training on first aid for pediatric trauma			**<0.001**		**0.006**		**<0.001**
Yes	300(63.56)	44[42,48.5]		36[32,39]		30.81 ± 4.98	
No	172(36.44)	41.5[37,44]		34[32,38]		25.94 ± 5.86	
Experience in organizing first aid for pediatric trauma			**<0.001**		0.857		**<0.001**
Yes	70(14.83)	44[43,52]		36[32,39]		32.02 ± 4.20	
No	402(85.17)	43[40,46]		35[32,38]		28.51 ± 5.89	
Teaching hospital			**<0.001**		**0.016**		**<0.001**
Yes	427(90.47)	44[41,47]		36[32,38]		29.43 ± 5.70	
No	14(2.97)	40[39,44]		32.5[32,35]		25.78 ± 4.42	
Not sure	31(6.57)	38[33,43]		32[31,38]		25.06 ± 6.02	
A hospital with standardized first aid procedures			**<0.001**		0.763		**<0.001**
Yes	429(90.89)	44[41,47]		36[32,38]		29.53 ± 5.54	
No	7(1.48)	39[31,45]		36[31,40]		29.42 ± 5.94	
Not sure	36(7.63)	37.5[32,40.5]		34.5[32,38]		23.08 ± 5.74	

### Distribution of responses to knowledge, attitude, and practice

The distribution of knowledge dimensions showed that the three questions with the highest number of participants choosing the “Not knowledgeable” and “Not knowledgeable at all” options were “Prehospital assessment tools for pediatric trauma include PTS, rapid trauma assessment, and physical examination.” (K2) with 17.37%, “After being transported to the hospital, trauma children should undergo in-hospital reassessment, which includes circulation assessment.” (K4.3) with 11.87%, and “Pediatric trauma assessments differ significantly from adult assessments in aspects such as airway, breathing, circulation, disability, exposure, and emotional considerations.” (K3) with 6.78% (Table 2).

**Table 2.** Distribution of knowledge dimension responses.

Knowledge	*n* (%)				
	Very knowledgeable	Knowledgeable	Unsure	Not knowledgeable	Not knowledgeable at all
1. Pediatric trauma includes physical injuries such as traffic accidents and falls, most of which are accidental injuries.	104(22.03)	288(61.02)	61(12.92)	19(4.03)	/
2. Prehospital assessment tools for pediatric trauma include PTS, rapid trauma assessment, and physical examination.	60(12.71)	207(43.86)	123(26.06)	77(16.31)	5(1.06)
3. Pediatric trauma assessments differ significantly from adult assessments in aspects such as airway, breathing, circulation, disability, exposure, and emotional considerations.	75(15.89)	282(59.75)	83(17.58)	31(6.57)	1(0.21)
4. After being transported to the hospital, trauma children should undergo in-hospital reassessment, which includes the following in addition to vital signs:					
4.1 Airway and breathing assessment	147(31.14)	267(56.57)	40(8.47)	18(3.81)	/
4.2 Circulation assessment	131(27.75)	271(57.42)	46(9.75)	24(5.08)	/
4.3 Burn assessment	69(14.62)	242(51.27)	105(22.25)	53(11.23)	3(0.64)
4.4 Pain assessment	82(17.37)	292(61.86)	80(16.95)	17(3.6)	1(0.21)
5. Prehospital first aid should quickly determine whether life-threatening injuries are present in trauma children, such as altered consciousness, airway obstruction, open pneumothorax, and massive hemorrhage.	98(20.76)	285(60.38)	64(13.56)	25(5.3)	/
6. Basic prehospital first aid measures include controlling bleeding, opening the airway, and performing cardiopulmonary resuscitation (CPR).	116(24.58)	294(62.29)	46(9.75)	15(3.18)	1(0.21)
7. According to prehospital assessment results, high-risk trauma children need to be quickly transferred to pediatric specialty hospitals or large comprehensive hospitals.	89(18.86)	294(62.29)	71(15.04)	18(3.81)	/
8. In-hospital emergency care primarily includes controlling bleeding, fluid resuscitation, management of traumatic brain injury, and pain treatment.	147(31.14)	267(56.57)	40(8.47)	18(3.81)	/

Responses to the attitude dimension showed that 53.81% strongly agreed and 41.74% agreed that medical personnel involved in pediatric trauma first aid often face greater psychological pressure (A5), 41.74% strongly agreed and 51.27% agreed that due to anatomical, physiological, and psychological differences between children and adults, pediatric trauma first aid is more challenging (A3), and 22.03% strongly agreed and 39.83% agreed that there is a lack of standardized pediatric trauma first aid guidelines (A2) (Table 3).

**Table 3.** Distribution of attitude dimension responses.

Attitude	Strongly agree	Agree	Neutral	Disagree	Strongly disagree
1. All pediatric nurses should receive training on trauma first aid. a. Strongly agree	243(51.48)	200(42.37)	27(5.72)	1(0.21)	1(0.21)
2. You believe that there is a lack of standardized pediatric trauma first aid guidelines.	104(22.03)	188(39.83)	147(31.14)	24(5.08)	9(1.91)
3. Due to anatomical, physiological, and psychological differences between children and adults, pediatric trauma first aid is more challenging.	197(41.74)	242(51.27)	30(6.36)	3(0.64)	/
4. Emergency care for pediatric trauma places higher professional demands on medical personnel.	244(51.69)	215(45.55)	13(2.75)	/	/
5. Medical personnel involved in pediatric trauma first aid often face greater psychological pressure.	254(53.81)	197(41.74)	20(4.24)	1(0.21)	/
6. Pediatric trauma first aid requires multidisciplinary team collaboration.	273(57.84)	184(38.98)	15(3.18)	/	/
7. Accurate assessment of the condition is critical for pediatric trauma first aid.	284(60.17)	175(37.08)	13(2.75)	/	/
8. The cooperation of parents should be sought whenever possible during pediatric trauma first aid.	244(51.69)	198(41.95)	28(5.93)	2(0.42)	/

Responses to the practice dimension showed that 28.6% rarely and 7.42% never participate in training on pediatric trauma first aid (P1), as well as 20.97% rarely and 1.91% never actively seek knowledge about pediatric trauma first aid (P2). Meanwhile, 12.92% disagreed, and 2.97% strongly disagreed that they can accurately use PTS during pediatric trauma assessments (P3.1) (Table 4).

**Table 4.** Distribution of practice dimension responses.

Practice	Always	Often	Sometimes	Rarely	Never
1. You regularly participate in training on pediatric trauma first aid.	47(9.96)	102(21.61)	153(32.42)	135(28.6)	35(7.42)
2. You actively seek knowledge about pediatric trauma first aid.	45(9.53)	145(30.72)	174(36.86)	99(20.97)	9(1.91)
3. During pediatric trauma assessments, you can accurately use the following tools:	**Strongly agree**	**Agree**	**Neutral**	**Disagree**	**Strongly disagree**
3.1 Pediatric Trauma Score(PTS)	74(15.68)	154(32.63)	169(35.81)	61(12.92)	14(2.97)
3.2 Rapid AVPU (Alert, Verbal, Pain, Unresponsive) Assessment	117(24.79)	202(42.8)	114(24.15)	30(6.36)	9(1.91)
3.3 Pediatric Modified Glasgow Coma Scale (GCS)	105(22.25)	208(44.07)	116(24.58)	33(6.99)	10(2.12)
3.4 FLACC Pain Scale	93(19.7)	180(38.14)	143(30.3)	42(8.9)	14(2.97)
4. You recommend involving parents as emotional support during patient care.	133(28.18)	203(43.01)	100(21.19)	29(6.14)	7(1.48)
5. When a patient requires transport, you contact the receiving hospital in advance to provide information about the patient’s medical history and condition.	223(47.25)	163(34.53)	65(13.77)	15(3.18)	6(1.27)

Further correlation analysis revealed positive correlations between knowledge scores and attitude scores (*r* = 0.410, *p* < 0.001), as well as between knowledge scores and practice scores (*r* = 0.655, *p* < 0.001). Additionally, attitude scores were positively correlated with practice scores (*r* = 0.388, *p* < 0.001). However, the correlations between PSS and the other three items were not significant (Table S1). The absence of significant linear correlations between PSS total scores and KAP outcomes underscores the value of latent profile analysis, which identified distinct subgroups with meaningful differences in KAP that would be obscured by traditional variable-centered approaches.

### LPA analysis and characteristics of participants across profiles

**Figure 1.**
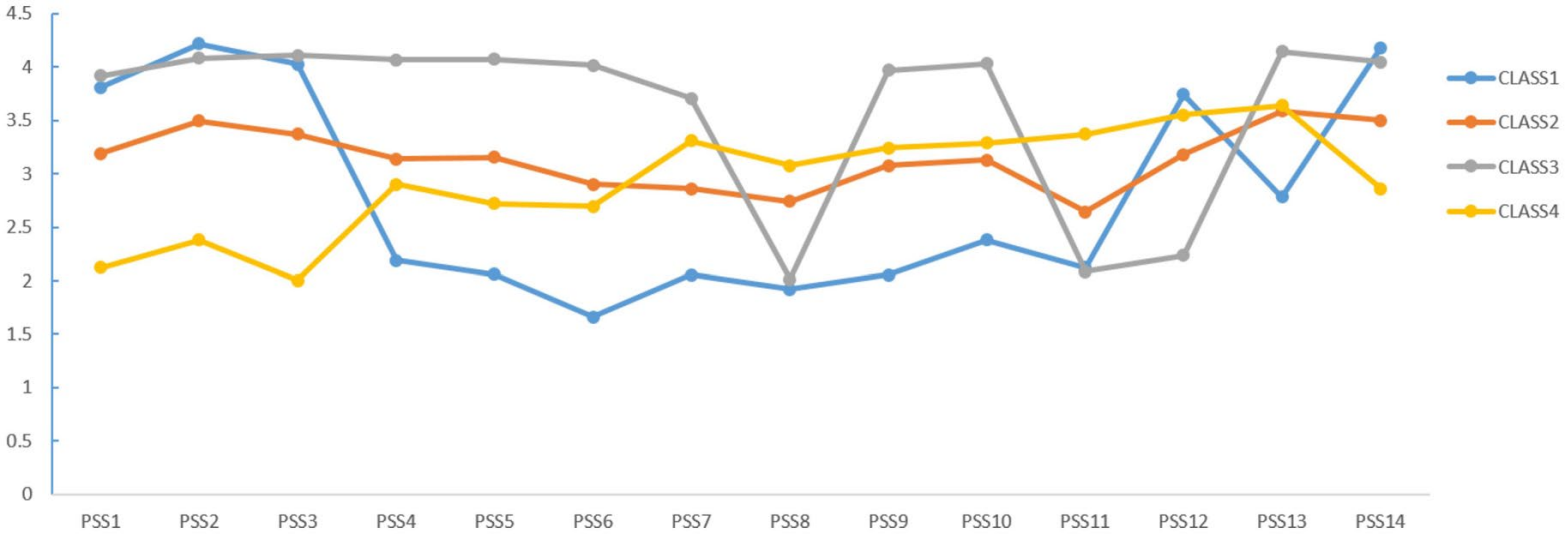
PSS-14 scores across profiles.

LPA was conducted using the PSS-14 scores (Figure 1) as manifest indicators. For the LPA, the individual subscale scores (perceived distress and perceived coping) were used rather than the total PSS-14 score to allow for the identification of distinct patterns characterized by different combinations of perceived distress levels and perceived coping. This analytical approach recognizes that individuals may experience varying levels of perceived distress alongside different perceived coping, yielding clinically meaningful profiles that would be obscured if only total perceived stress scores were examined. Models with 1 to 5 latent profiles were fitted, and the best-fitting model was determined based on fit indices, interpretability, and profile distribution (Table S2). The four-profile solution was selected as the optimal model, supported by lower values compared to the other-profile models. The four-profile model also demonstrated a high entropy value (0.900), indicating good classification accuracy. Both the LMR and BLRT were significant (*p* = 0.0298 and *p* < 0.001), supporting the addition of a fourth profile. The four latent profiles identified and their respective proportions were as follows: Profile 1 (high perceived distress + high perceived coping): 125 individuals (26.48%); Profile 2 (moderate perceived distress + moderate perceived coping): 226 individuals (47.88%); Profile 3 (moderate perceived distress + low perceived coping): 72 individuals (15.25%); Profile 4 (low perceived distress + moderate perceived coping): 49 individuals (10.38%). These profiles provide a meaningful interpretation of perceived stress and coping patterns in the study population, with Profile 2 being the largest group and Profile 4 the smallest.

The four latent profiles were classified based on the PSS-14 scores, which measured perceived stress dimensions. Statistical comparisons revealed significant differences in KAP scores across the four profiles (all *p* < 0.001), as well as in demographic characteristics (Table 5). Profile 1 exhibited the highest knowledge and attitude scores, demonstrating that nurses with high perceived coping maintained more favorable self-reported knowledge and attitude responses despite elevated perceived stress levels. For knowledge (Ksum), Profile 1 had the highest median score [44 (42, 49)], followed by Profile 2. Attitude (Asum) in Profile 1 also reached the highest median score [37 (33, 39)], indicating an association between high perceived coping and higher self-reported practice, even when perceived distress is elevated. Practice (Psum) in Profile 1 was similarly high [28 (25, 34)] but was lower than in the other profiles. Participants in Profile 1 were also the oldest, with a median age of 35 years [31,38], and had the highest proportion of females (98.4%). In terms of perceived stress, Profile 1 exhibited the highest perceived distress (measured by negatively worded items: [24 (23, 25)], where higher scores indicate higher perceived distress) and the highest perceived coping (measured by positively worded items: [15 (13, 17)], where lower scores indicate greater perceived coping). Profile 2 showed moderate knowledge, attitude, and practice scores. Knowledge (Ksum) and attitude (Asum) were ranked second [43 (40, 45) and 34 (32, 38), respectively]. For practice (Psum), Profile 2 scored [28 (25, 32)], showing mid-range values.

**Table 5.** Participant’s characteristics and KAP scores across profiles.

Variable	Profile-1 *N* = 125	Profile-2 *N* = 226	Profile-3 *N* = 72	Profile-4 *N* = 49	*P*
Knowledge scores	44[42,49]	43[40,45]	44[41,48.5]	43[39,46]	<0.001
Attitude scores	37[33,39]	34[32,38]	36[32,40]	35[32,38]	<0.001
Practice scores*	28[25,34]	28[25,32]	30.5[28,36]	29[26,34]	<0.001
Perceived stress	24[23,25]	22[21,23]	22[21,23.5]	19[18,20]	<0.001
Perceived coping	15[13,17]	22[20,23]	28[27,29]	22[20,24]	<0.001
Age, years	35[31,38]	35[32,37]	34[31.5,38]	34[30,38]	<0.001
Gender					0.030^a^
Male	2(1.6)	10(4.42)	8(11.11)	2(4.08)	
Female	123(98.4)	216(95.58)	64(88.89)	47(95.92)	
Education					0.340 ^a^
Associate degree or below	5(4)	10(4.42)	7(9.72)	1(2.04)	
Bachelor’s degree	116(92.8)	208(92.04)	63(87.5)	44(89.8)	
Master’s degree	4(3.2)	8(3.54)	2(2.78)	4(8.16)	
Doctorate	**/**	**/**	**/**	**/**	
Professional title					0.387 ^a^
Junior level	41(32.8)	81(35.84)	32(44.44)	17(34.69)	
Intermediate level	78(62.4)	129(57.08)	35(48.61)	30(61.22)	
Senior level	3(2.4)	9(3.98)	**/**	1(2.04)	
No professional title	3(2.4)	7(3.1)	5(6.94)	1(2.04)	
Monthly income, CNY					-^a^
<5000	15(12)	33(14.6)	16(22.22)	11(22.45)	
5000–10,000	96(76.8)	151(66.81)	48(66.67)	29(59.18)	
10,000–20,000	12(9.6)	24(10.62)	4(5.56)	7(14.29)	
>20,000	/	/	/	/	
Prefer not to disclose	2(1.6)	18(7.96)	4(5.56)	2(4.08)	
Working experience, years	12[8,15]	11[9,14]	11[7,14]	11[7,14]	0.822
Experience of prehospital first aid for pediatric trauma					0.050
Yes	19(15.2)	38(16.81)	18(25)	15(30.61)	
No	106(84.8)	188(83.19)	54(75)	34(69.39)	
Experience of first aid for pediatric trauma					0.849
Yes	49(39.2)	80(35.4)	27(37.5)	20(40.82)	
No	76(60.8)	146(64.6)	45(62.5)	29(59.18)	
Experience of Training on first aid for pediatric trauma					0.550
Yes	85(68)	141(62.39)	42(58.33)	32(65.31)	
No	40(32)	85(37.61)	30(41.67)	17(34.69)	
Experience in organizing first aid for pediatric trauma					0.318
Yes	19(15.2)	28(12.39)	12(16.67)	11(22.45)	
No	106(84.8)	198(87.61)	60(83.33)	38(77.55)	
Teaching hospital					0.708 ^a^
Yes	116(92.8)	200(88.5)	66(91.67)	45(91.84)	
No	4(3.2)	7(3.1)	1(1.39)	2(4.08)	
Not sure	5(4)	19(8.41)	5(6.94)	2(4.08)	
A hospital with standardized first aid procedures					0.673 ^a^
Yes	115(92)	202(89.38)	65(90.28)	47(95.92)	
No	1(0.8)	6(2.65)	/	/	
Not sure	9(7.2)	18(7.96)	7(9.72)	2(4.08)	

Note: For perceived stress items (negatively worded), higher scores indicate higher stress. For perceived coping items (positively worded), lower scores indicate better coping ability.

*: The practice were skewed distributed when grouped according to the latent profiles, and were compared using Kruskal-Wallis test.

^a^: Categorical data were compared using the chi-squared test or Fisher’s exact test, as appropriate.

Profile 2’s age distribution was similar to Profile 1, with a median of 35 years [32,37]. Profile 2 also had a high proportion of females (95.58%), but slightly fewer than Profile 1. The perceived distress levels (PSSpart1) were moderate, with Profile 2 showing perceived distress [22 (21, 23)] and moderate perceived coping [22 (20, 23)]. Profile 3 exhibited moderate knowledge [44 (41, 48.5)] and attitude [36 (32, 40)], but the highest practice scores [30.5 (28, 36)].

Profile 3 had a similar age distribution to Profile 2, with a median of 34 years [31.5, 38], and a higher proportion of males (11.11%). Perceived stress levels were moderate to high, with Profile 3 reporting high perceived distress [22 (21, 23.5)] but the lowest perceived coping[28 (27, 29)]. Profile 4 demonstrated relatively lower knowledge and attitude scores compared to other profiles, though practice scores were comparable to Profile 2. Knowledge (Ksum) had a median of [43 (39, 46)], similar to Profile 2’s score of [43 (40, 45)], while attitude (Asum) was [35 (32, 38)]. Practice (Psum) was [29 (26, 34)], comparable to Profile 2’s [28 (25, 32)]. This pattern suggests that low perceived distress combined with moderate perceived coping may be particularly disadvantageous for knowledge acquisition and positive attitudes, despite not substantially impairing practice behaviors. Profile 4 had a median age of 34 years, with a similar gender distribution to Profile 2 (95.92% females). This profile exhibited the lowest perceived distress [19 (18, 20)] with moderate perceived coping [22 (20, 24)] (Table 5). The significant differences in KAP scores across perceived stress profiles demonstrate that patterns of perceived distress and perceived coping are meaningfully associated with practice. Specifically, profiles characterized by high perceived coping (Profiles 1) demonstrated superior knowledge and attitude scores compared to profiles with lower perceived coping (Profile 3), regardless of perceived distress levels.

## Discussion

This study yielded several significant findings regarding pediatric nurses’ perceived stress profiles and KAP towards first-aid for pediatric trauma. Latent profile analysis identified four distinct perceived stress patterns among pediatric nurses, characterized by varying combinations of perceived distress and perceived coping. The profile with high perceived coping, despite elevated perceived stress levels, consistently demonstrated higher scores across all KAP domains. Furthermore, strong positive correlations emerged between knowledge, attitudes, and practice scores, indicating these components are deeply interconnected in developing practice. The study also revealed specific areas requiring improvement, particularly in prehospital assessment tools and procedures, while highlighting the crucial role of psychological resilience in maintaining practice.

These findings align with and extend current understanding in several important ways. The identification of distinct perceived stress profiles supports recent research highlighting the complex nature of healthcare workers’ stress responses [21,22]. The NICU work environment influences professional well-being, which can influence the quality of care [23]. Our observation that nurses with higher perceived coping reported higher knowledge and attitude scores regardless of their perceived distress levels suggests that perceived coping abilities may be more strongly associated with self-reported practice than perceived distress alone. This finding aligns with the growing body of evidence emphasizing psychological resilience in healthcare settings, though the causal relationships and mechanisms require further investigation through longitudinal and experimental research [24,25]. One possible explanation for the association between perceived coping and higher knowledge and attitude scores may involve preservation of cognitive resources. If perceived coping does buffer the effects of perceived distress (a hypothesis requiring experimental validation), this mechanism may involve perceived coping buffering stress effects, which may involve preservation of cognitive resources. High perceived coping ability may enable nurses to maintain situational awareness, working memory capacity, and decision-making accuracy even under conditions of high perceived distress. When individuals appraise themselves as capable of handling stressors, they may allocate cognitive resources more efficiently, preventing the cognitive overload that typically impairs practice under stress. This interpretation is consistent with cognitive load theory and suggests that interventions targeting coping skills could protect cognitive function during critical tasks. In the context of pediatric trauma care, where rapid assessment, accurate calculations (e.g. weight-based medication dosing), and multitasking are essential, preserving cognitive resources through enhanced perceived coping becomes particularly crucial for patient safety and optimal outcomes. This interpretation is further supported by recent research demonstrating a direct relationship between emotional intelligence and adaptive coping behaviors among NICU nurses [26]. That study found that nurses with higher emotional intelligence employed more effective coping strategies, which may explain why Profile 1 nurses in our study, despite experiencing high perceived distress, maintained superior knowledge and attitude scores through effective perceived coping. Emotional intelligence may facilitate the appraisal of stressors as manageable rather than overwhelming, thereby preserving the cognitive resources necessary for complex clinical tasks. This finding has particular significance for pediatric trauma care, where the emotional demands of treating young patients can intensify workplace stress. The variation in perceived distress profiles suggests that individual differences in distress response and coping mechanisms play a crucial role in attitudes and practice, beyond what might be expected from traditional measures of experience or training alone.

Nevertheless, this study also indicated that nurses in Profile 3 (moderate perceived distress and low perceived coping) exhibited relatively lower knowledge and attitude scores compared to profile 1, but the highest practice scores. This discrepancy may be explained by cognitive biases from the self-reported data, specifically the Dunning-Kruger effect, where individuals with inadequate actual knowledge or competence lack the ability to recognize their deficits, leading to an overestimation of practice score [27,28]. A repressive and defensive coping style could also be a possible explanation. Nurses with lower perceived coping abilities may feel more vulnerable to professional threats in high-stress environments and, consequently, inflate their self-reported practice scores as a compensatory strategy [29].

In contrast, this study revealed that nurses in Profile 1 (high perceived distress combined with high perceived coping) consistently achieved the highest knowledge and attitude scores, despite reporting the greatest perceived distress of all four profiles, together with high practice scores (but lower practice scores than in profiles 3 and 4). This pattern is consistent with the transactional model of stress, in which a stressor accompanied by sufficient perceived coping resources is appraised as a challenge rather than a threat; such challenge appraisals have been associated with more regulated psychophysiological responses and superior practice under high-pressure conditions [30]. In line with the job demand-resources perspective, both task demands and personal resources can independently and positively influence knowledge acquisition performance and attitudes, so that individuals who simultaneously perceive high demands and high resources are well positioned to convert stress into effective clinical engagement [31]. Self-efficacy may further account for this advantage, as it functions as a key personal resource that buffers the adverse effects of stressors on nurses’ performance and supports the acquisition of clinical knowledge [32]. Notably, this buffering effect appears most pronounced among older nurses in supervisory or leadership roles [33], consistent with the observation that Profile 1 comprised the oldest participants, potentially reflecting accumulated experience and consolidated coping repertoires that sustain high KAP levels even under elevated stress.

The strong associations found between knowledge, attitudes, and practices domains support existing literature indicating these components reinforce each other in clinical skill development [34]. This interconnection suggests that interventions targeting any single component may have ripple effects across all domains, potentially amplifying the impact of training initiatives. The relationship between experience and practice proficiency, moderated by perceived coping abilities, aligns with literature emphasizing both technical and psychological aspects of practice [35]. This finding underscores the importance of developing integrated approaches to professional development that address both technical skills and psychological resilience.

An important methodological finding of this study is the absence of significant linear correlations between overall PSS scores and KAP outcomes, which might suggest that no relationship exists between perceived stress and practice. However, the latent profile analysis revealed substantial and meaningful differences in KAP scores across distinct perceived distress profiles, demonstrating that the relationship between stress and higher KAP scores is more nuanced than simple linear associations would suggest. By identifying subgroups with distinct patterns of perceived distress and perceived coping, and then comparing KAP outcomes across these subgroups, we were able to detect associations that would be obscured by traditional variable-centered correlational approaches.

Regarding knowledge gaps in specialized assessment tools, our findings parallel previous studies identifying similar deficits as barriers to effective trauma care delivery [36]. However, our research extends beyond previous work by demonstrating that these knowledge gaps are not uniformly distributed across perceived stress profiles, suggesting that psychological factors may influence both the acquisition and application of technical knowledge. This observation has significant implications for how training programs should be designed and implemented, particularly in high-stress pediatric emergency settings.

The study revealed several critical insights about the relationship between perceived distress profiles and KAP scores that warrant further discussion. First, the finding that nurses with better perceived coping abilities maintained higher KAP levels, regardless of their absolute stress levels, challenges traditional approaches to stress management in healthcare settings [37,38]. This suggests that resilience-building strategies may be more effective than stress-reduction interventions alone. Second, the variation in KAP scores across distress profiles indicates that psychological factors may influence learning and skill application more significantly than previously recognized.

Our results suggest that psychological factors, including perceived coping abilities, are associated with self-reported practice. Previous research has demonstrated that supportive learning environments and confidence-building exercises can improve engagement and performance in trauma care settings, suggesting potential intervention targets that warrant investigation in future studies with pediatric nurses [39]. This finding has particular relevance for pediatric trauma care, where the emotional stakes are often higher than in adult care settings. The study suggests that creating supportive work environments that acknowledge and address the psychological demands of pediatric trauma care could enhance both individual and team practice. Furthermore, the findings indicate that regular assessment of both clinical knowledge and psychological readiness may help identify nurses requiring additional support or targeted interventions.

This study has several limitations that should be acknowledged. First, the cross-sectional design prevents us from establishing causal relationships between perceived distress profiles and KAP scores, limiting the ability to determine the direction of these associations. The cross-sectional design also prevents examination of how perceived distress profiles and KAP evolve over time; longitudinal studies are needed to establish temporal relationships and identify whether interventions can facilitate transitions from less adaptive to more adaptive perceived distress profiles. Second, the sample consisted predominantly of female nurses (95.34%), which may reduce the generalizability of the findings to male pediatric nurses or other healthcare professionals. In addition, convenience sampling may limit generalizability. Third, the PSS-14 demonstrated acceptable but modest internal consistency (*α* = 0.7312) in our pilot sample, which may have attenuated some associations between perceived stress dimensions and KAP outcomes. Furthermore, PSS-14 measures general perceived distress rather than occupational stress. A more refined assessment tool could be necessary in future studies. Fourth, data were collected from a single province (Henan) in China, limiting generalizability to other regions with different healthcare systems, resource availability, training standards, and cultural contexts. The findings may not apply to settings with different nurse-to-patient ratios, trauma volumes, or organizational support structures. Fifth, the study relied on self-reported KAP measures, including Likert-scale assessments of self-perceived knowledge, which are inherently subject to social desirability bias and may overestimate actual practice. This distinction is important because self-perceived knowledge may not accurately reflect actual knowledge; individuals may overestimate their knowledge (Dunning-Kruger effect) or, conversely, highly knowledgeable individuals may underestimate their expertise. The identified gaps in self-perceived knowledge (e.g. regarding PTS assessment tools) suggest areas where nurses feel unprepared, which is valuable for identifying training needs, but actual knowledge deficits require objective assessment to confirm. Future studies should incorporate objective evaluation methods, such as validated knowledge tests with correct/incorrect responses, simulation-based assessments, standardized patient scenarios, and direct observation of trauma care delivery, to provide a more accurate and comprehensive assessment of practice. Sixth, the use of convenience sampling and online questionnaire distribution may have introduced selection bias, potentially favoring nurses with better computer skills, more available time, or higher motivation to participate. Seventh, all variables were collected using self-report measures administered at the same time point, raising the possibility of common method variance inflating the observed relationships among constructs. Future research employing longitudinal designs, more diverse and representative samples, multi-method assessment approaches, and objective practice measures is needed to address these limitations and validate our findings across different contexts.

## Conclusions

These findings highlight the need for tailored interventions that address both the psychological well-being and educational needs of pediatric nurses. The present findings may have implications for hospital management, depending upon local practices and policies already in place: (1) implement regular screening using validated instruments like the PSS-14 to identify nurses with low perceived coping abilities (e.g. Profile 3: moderate perceived distress with low perceived coping) who may benefit from targeted psychological support; (2) integrate evidence-based stress management techniques such as mindfulness-based stress reduction (MBSR), cognitive-behavioral strategies, or structured debriefing sessions into continuing education programs for pediatric trauma care; (3) develop mentorship or peer support programs pairing nurses with high perceived coping abilities (Profile 1) with those in lower-performing profiles to facilitate knowledge transfer and coping strategy sharing; (4) design training programs that simultaneously address first-aid practices (particularly prehospital assessment tools like PTS) and stress resilience skills, recognizing their interconnected nature; and (5) evaluate the impact of these interventions on both nurse psychological well-being and patient care outcomes through longitudinal follow-up studies. By considering these evidence-based strategies, healthcare institutions may foster a more resilient workforce capable of delivering optimal care in high-pressure pediatric trauma situations.

## Supplementary Material

Questionnaire.docx

Supplementary tables.docx

## Data Availability

All data generated or analyzed during this study are included in this published article.
