# Supplementary material for: Pediatric nurses’ stress and their knowledge, attitudes, and practices towards first-aid for pediatric trauma: a latent profile analysis
Source: Ann Med. 2026 Jul 4;58(1):2696066. doi: 10.1080/07853890.2026.2696066 (PMC13347846; doi:10.1080/07853890.2026.2696066)
Supplement: Questionnaire.docx [file IANN_A_2696066_SM6429.docx]

| Dear Participant：  We are researchers from *Hospital, and we sincerely invite you to participate in our study. This research aims to understand your knowledge, attitudes, and willingness to practice in the field of pediatric trauma first aid. The findings will serve as a basis for developing scientific intervention strategies, which may help more people and improve their health in the future. Your participation in this study is entirely voluntary. If you agree to take part, please read the following instructions carefully:  1.Please complete the questionnaire. There are no right or wrong answers; simply respond based on your actual situation. If you encounter any questions during the process, feel free to reach out to us. After completing the questionnaire, please submit it promptly.  2.This study involves a simple survey and will not pose any physical or psychological harm to you. However, it may include some personal information, such as your gender and age. We will strictly protect your privacy and ensure the confidentiality of your information. Please feel free to complete the questionnaire.  3.As a participant, you may request information and updates related to this research at any time. If you decide to withdraw from the study, please inform us, and your data will not be included in the research results.  Finally, we sincerely thank you for taking the time to support our scientific research!  □I have been informed and agree to the use of the collected data for scientific research.  Informed Consent Signature:  Date of Participation: ______ Year ______ Month ______ Day |
| --- |

| **Part I-Basic Information** | |
| --- | --- |
| 1. **Your age：** | Years old |
| **2. Your gender** | a. Male  b. Female |
| **3. Education level：** | a. Associate degree or below b. Bachelor's degree c. Master's degree d. Doctorate |
| **4. Professional title：** | a. Junior level b. Intermediate level c. Senior level d. No professional title |
| **5. Your average monthly income over the past year: ______ yuan** | a.<5000  b.5000-10000  c.10000-20000  d.>20000  e. Prefer not to disclose |
| **6.Year of work experience** | years |
| **7.Have you participated in pre-hospital first aid for pediatric trauma patients?** | a. Yes b. No |
| **7-1.In the past year, how many pre-hospital pediatric trauma cases did you participate in on average per month?** | a. Fewer than 10 cases b. 10–50 cases c. 50–100 cases d. 100–200 cases e. More than 200 cases |
| **8.Have you participated in emergency treatment for pediatric trauma patients?** | a. Yes b. No |
| **8-1.In the past year, how many pediatric trauma emergency cases did you participate in on average per month?** | a. Fewer than 10 cases b. 10–50 cases c. 50–100 cases d. 100–200 cases e. More than 200 cases |
| **9.Have you ever attended training on pediatric trauma first aid?** | a. Yes b. No |
| **10.Have you organized pediatric trauma first aid?** | a. Yes b. No |
| **11.What is the grade of your hospital?** | a. Tertiary hospital b. Secondary hospital c. Primary hospital d. Other |
| **12.Is your hospital a teaching hospital?** | a. Yes b. No c. Not sure |
| **13. Professional title** | a. No professional title b. Junior level c. Intermediate level d. Senior level |
| **14.Does your hospital have standardized pre-hospital/in-hospital first aid procedures?** | a. Yes b. No c. Not sure |

**Part II-Knowledge**

**Please select one option based on your understanding of the question, ranging from "Very Knowledgeable" to "Not Knowledgeable at All.”**

| **1.Pediatric trauma includes physical injuries such as traffic accidents and falls, most of which are accidental injuries.** | **a. very knowledgeable** | **b. knowledgeable** | **c. unsure** | **d. not knowledgeable** | **e. not knowledgeable at all** |
| --- | --- | --- | --- | --- | --- |
| **2.Pre-hospital assessment tools for pediatric trauma include PTS, rapid trauma assessment, and physical examination.** | **a. very knowledgeable** | **b. knowledgeable** | **c. unsure** | **d. not knowledgeable** | **e. not knowledgeable at all** |
| **3.Pediatric trauma assessments differ significantly from adult assessments in aspects such as airway, breathing, circulation, disability, exposure, and emotional considerations.** | **a. very knowledgeable** | **b. knowledgeable** | **c. unsure** | **d. not knowledgeable** | **e. not knowledgeable at all** |
| **4.After being transported to the hospital, trauma children should undergo in-hospital reassessment, which includes the following in addition to vital signs:** |  |  |  |  |  |
| **Airway and breathing assessment** | **a. very knowledgeable** | **b. knowledgeable** | **c. unsure** | **d. not knowledgeable** | **e. not knowledgeable at all** |
| **Circulation assessment** | **a. very knowledgeable** | **b. knowledgeable** | **c. unsure** | **d. not knowledgeable** | **e. not knowledgeable at all** |
| **Burn assessment** | **a. very knowledgeable** | **b. knowledgeable** | **c. unsure** | **d. not knowledgeable** | **e. not knowledgeable at all** |
| **Pain assessment** | **a. very knowledgeable** | **b. knowledgeable** | **c. unsure** | **d. not knowledgeable** | **e. not knowledgeable at all** |
| **5.Pre-hospital first aid should quickly determine whether life-threatening injuries are present in trauma children, such as altered consciousness, airway obstruction, open pneumothorax, and massive hemorrhage.** | **a. very knowledgeable** | **b. knowledgeable** | **c. unsure** | **d. not knowledgeable** | **e. not knowledgeable at all** |
| **6.Basic pre-hospital first aid measures include controlling bleeding, opening the airway, and performing cardiopulmonary resuscitation (CPR).** | **a. very knowledgeable** | **b. knowledgeable** | **c. unsure** | **d. not knowledgeable** | **e. not knowledgeable at all** |
| **7.According to pre-hospital assessment results, high-risk trauma children need to be quickly transferred to pediatric specialty hospitals or large comprehensive hospitals.** | **a. very knowledgeable** | **b. knowledgeable** | **c. unsure** | **d. not knowledgeable** | **e. not knowledgeable at all** |
| **8.In-hospital emergency care primarily includes controlling bleeding, fluid resuscitation, management of traumatic brain injury, and pain treatment.** | **a. very knowledgeable** | **b. knowledgeable** | **c. unsure** | **d. not knowledgeable** | **e. not knowledgeable at all** |

**Part-III Attitude**

**Please indicate your agreement with the following statements by selecting one option ranging from "Strongly Agree" to "Strongly Disagree.”**

| **1.All pediatric nurses should receive training on trauma first aid. a. Strongly agree** | **a. strongly agree** | **b. agree** | **c. neutral** | **d. disagree** | **e. strongly disagree** |
| --- | --- | --- | --- | --- | --- |
| **2.You believe that there is a lack of standardized pediatric trauma first aid guidelines.** | **a. strongly agree** | **b. agree** | **c. neutral** | **d. disagree** | **e. strongly disagree** |
| **3.Due to anatomical, physiological, and psychological differences between children and adults, pediatric trauma first aid is more challenging.** | **a. strongly agree** | **b. agree** | **c. neutral** | **d. disagree** | **e. strongly disagree** |
| **4.Emergency care for pediatric trauma places higher professional demands on medical personnel.** | **a. strongly agree** | **b. agree** | **c. neutral** | **d. disagree** | **e. strongly disagree** |
| **5.Medical personnel involved in pediatric trauma first aid often face greater psychological pressure.** | **a. strongly agree** | **b. agree** | **c. neutral** | **d. disagree** | **e. strongly disagree** |
| **6.Pediatric trauma first aid requires multidisciplinary team collaboration.** | **a. strongly agree** | **b. agree** | **c. neutral** | **d. disagree** | **e. strongly disagree** |
| **7.Accurate assessment of the condition is critical for pediatric trauma first aid.** | **a. strongly agree** | **b. agree** | **c. neutral** | **d. disagree** | **e. strongly disagree** |
| **8.The cooperation of parents should be sought whenever possible during pediatric trauma first aid.** | **a. strongly agree** | **b. agree** | **c. neutral** | **d. disagree** | **e. strongly disagree** |

**Part IV- Practice**

**Please choose the option that best matches your actions in the given situations.**

| **1.You regularly participate in training on pediatric trauma first aid.** | **a. always** | **b. often** | **c. sometimes** | **d. rarely** | **e. never** |
| --- | --- | --- | --- | --- | --- |
| **2.You actively seek knowledge about pediatric trauma first aid.** | **a. always** | **b. often** | **c. sometimes** | **d. rarely** | **e. never** |
| **3.During pediatric trauma assessments, you can accurately use the following tools:** |  |  |  |  |  |
| **Pediatric Trauma Score（PTS）** | **a. strongly agree** | **b. agree** | **c. neutral** | **d. disagree** | **e. strongly disagree** |
| **Rapid AVPU (Alert, Verbal, Pain, Unresponsive) Assessment** | **a. strongly agree** | **b. agree** | **c. neutral** | **d. disagree** | **e. strongly disagree** |
| **Pediatric Modified Glasgow Coma Scale (GCS)** | **a. strongly agree** | **b. agree** | **c. neutral** | **d. disagree** | **e. strongly disagree** |
| **FLACC Pain Scale** | **a. strongly agree** | **b. agree** | **c. neutral** | **d. disagree** | **e. strongly disagree** |
| **4.You recommend involving parents as emotional support during patient care.** | **a. always** | **b. often** | **c. sometimes** | **d. rarely** | **e. never** |
| **5.When a patient requires transport, you contact the receiving hospital in advance to provide information about the medical history and condition.** | **a. always** | **b. often** | **c. sometimes** | **d. rarely** | **e. never** |

**Part V - Perceived Stress Scale**

| 1.Felt upset because of something unexpected. | Never | Rarely | Sometimes | Often | Always |
| --- | --- | --- | --- | --- | --- |
| 2.Felt unable to control the important things in your life. | Never | Rarely | Sometimes | Often | Always |
| 3.Felt nervous, anxious, or under stress. | Never | Rarely | Sometimes | Often | Always |
| 4.Successfully dealt with irritating life hassles. | Never | Rarely | Sometimes | Often | Always |
| 5.Felt that you were effectively coping with important changes in your life. | Never | Rarely | Sometimes | Often | Always |
| 6.Felt confident in your ability to handle personal problems. | Never | Rarely | Sometimes | Often | Always |
| 7.Felt that things were going your way. | Never | Rarely | Sometimes | Often | Always |
| 8.Found yourself unable to deal with all the things you had to do. | Never | Rarely | Sometimes | Often | Always |
| 9.Been able to control irritating matters in your life. | Never | Rarely | Sometimes | Often | Always |
| 10.Felt that you were in control of your life. | Never | Rarely | Sometimes | Often | Always |
| 11.Felt angry because of things beyond your control. | Never | Rarely | Sometimes | Often | Always |
| 12.Often thought about tasks you had to complete. | Never | Rarely | Sometimes | Often | Always |
| 13.Often managed your time effectively. | Never | Rarely | Sometimes | Often | Always |
| 14.Felt overwhelmed by difficulties piling up, unable to overcome them. | Never | Rarely | Sometimes | Often | Always |
