# Supplementary material for: Pediatric nurses’ stress and their knowledge, attitudes, and practices towards first-aid for pediatric trauma: a latent profile analysis
Source: Ann Med. 2026 Jul 4;58(1):2696066. doi: 10.1080/07853890.2026.2696066 (PMC13347846; doi:10.1080/07853890.2026.2696066)
Supplement: Supplementary tables.docx [file IANN_A_2696066_SM6428.docx]

**Table S1. Correlation analysis**

|  | **Knowledge** | **Attitude** | **Practice** | **PSS** |
| --- | --- | --- | --- | --- |
| **Knowledge** | 1 |  |  |  |
| **Attitude** | 0.410 (P＜0.001) | 1 |  |  |
| **Practice** | 0.655 (P＜0.001) | 0.388 (P＜0.001) | 1 |  |
| **PSS** | -0.085(P=0.064) | -0.063(P=0.170) | 0.035(P=0.454) | 1 |

**Table S2. Latent Profile Analysis**

| **Model** | **AIC** | **BIC** | **aBIC** | **Entropy** | **LMR** | **BLRT** |
| --- | --- | --- | --- | --- | --- | --- |
| **1** | 17219.580 | 17335.975 | 17247.108 |  |  |  |
| **2** | 16095.553 | 16274.303 | 16137.829 | 0.890 | <0.001 | <0.001 |
| **3** | 15429.605 | 15670.709 | 15486.628 | 0.880 | 0.0992 | <0.001 |
| **4** | 15180.519 | 15483.979 | 15252.289 | 0.900 | 0.0298 | <0.001 |
| **5** | 14992.004 | 15357.818 | 15078.521 | 0.891 | 0.1237 | <0.001 |
